# Steroid Rebound Phenomenon as a Cause of Delayed Cerebral Vasospasm in Post-Splenectomy Pneumococcal Meningitis: A Case Report

**DOI:** 10.7759/cureus.33439

**Published:** 2023-01-06

**Authors:** Daiki Yokoyama, Hajime Ikenouchi, Tatsuo Miyamoto, Naoki Yamamoto, Kaoru Endo

**Affiliations:** 1 Department of Neurology, Sendai City Hospital, Sendai, JPN

**Keywords:** cerebral infarction, vasospasm, overwhelming post-splenectomy infection, pneumococcal meningitis, steroid rebound phenomenon

## Abstract

Pneumococcal meningitis as an overwhelming post-splenectomy infection (OPSI) has a higher risk of neurological complications and is sometimes life-threatening. In acute pneumococcal meningitis, four days of dexamethasone is widely used for the prevention of neurological complications. Herein, we report a 68-year-old woman with the diagnosis of pneumococcal meningitis as OPSI. With adequate antibiotics and dexamethasone, her symptoms gradually improved. However, after dexamethasone withdrawal, her consciousness got worse and got into a coma. Brain magnetic resonance imaging revealed acute cerebral infarctions in the bilateral middle cerebral artery territory with multiple vascular stenoses and hydrocephalus. Vascular stenoses improved by follow-up, suggesting cerebral vasospasm. There were no suggestive findings of cerebral vasculitis. Follow-up cerebrospinal fluid analysis showed remained pleocytosis with no bacteria, which could not suggest meningitis recurrence. Since steroid therapy was rapidly withdrawn, we diagnosed that the cerebral vasospasm was due to the steroid rebound phenomenon. The steroid rebound phenomenon due to the excessive immune response to bacterial microstructures has been reported in pneumococcal meningitis. Especially, the present case was asplenia and the usual dexamethasone use would not adequately suppress the immune response to bacterial microstructures. Since pneumococcal meningitis as OPSI has a higher risk of neurological complications, clinicians should consider longer and more cautious steroid tapering.

## Introduction

Pneumococcal meningitis as an overwhelming post-splenectomy infection (OPSI) has a higher risk of neurological complications and is sometimes life-threatening [1-3]. Pneumococcal meningitis is sometimes accompanied by neurological complications such as vasculitis, and cerebral vasospasm, resulting in cerebral infarctions [3]. In acute pneumococcal meningitis, four days of dexamethasone is widely used to prevent neurological complications [4]. In the previous reports, delayed neurological complications have been reported after initial treatment, especially after dexamethasone withdrawal, which has been described as “steroid rebound phenomenon” [5-7]. Here we report a case of delayed neurological complications by usual dexamethasone use in pneumococcal meningitis as OPSI.

## Case presentation

A 68-year-old woman was brought to our hospital because of fever and progressive consciousness disturbance over four days. She had a history of hypertension, diabetes, and took antihypertensive agents and oral hypoglycemic agents. She also had a history of gastrectomy and splenectomy for gastric cancer in her 50s. She has not been received pneumococcal vaccination. She had no sick contact with *Streptococcus pneumoniae.* She also had no family history of cancer, cerebrovascular, cardiovascular, or neurological diseases. At the presentation, her vital signs were as follows: systolic blood pressure 150 mmHg and diastolic blood pressure 100 mmHg, heart rate 100 bpm, respiratory rate 20 bpm, and body temperature 39.0°C. On the physical examination, there were no abnormal cardiac and breath sounds, no abnormalities in the abdomen, and no skin rash on the extremities. On the neurological examination, she had moderate consciousness disturbance (Glasgow coma scale of 11; E3V3M5) and showed positive meningeal signs. Laboratory tests showed a slightly elevated white blood cell count, decreased platelet count, slightly prolonged prothrombin time-international normalized ratio, elevated D-dimer level, elevated aspartate aminotransferase, decreased K level, and elevated C-reactive protein level. The additional blood test for autoimmune antibodies such as anti-nuclear antibodies, anti-neutrophil cytoplasmic antibody, SS-A/B antibody, and antiphospholipid antibody were negative (Table 1). 

**Table 1:** Laboratory findings of the present case Laboratory tests showed a slightly elevated white blood cell count, decreased platelet count, slightly prolonged prothrombin time-international normalized ratio, elevated D-dimer level, elevated aspartate aminotransferase, decreased K level, and elevated C-reactive protein level. The additional blood test for autoimmune antibodies such as anti-nuclear antibodies, anti-neutrophil cytoplasmic antibody, SS-A/B antibody, and antiphospholipid antibody were negative. Abbreviations: WBC, white blood cell; Hb, hemoglobin; Plt, platelets; PT-INR, prothrombin time-international normalized ratio; APTT, activated partial thromboplastin time; AST, aspartate aminotransferase; ALT, alanine aminotransferase; γGTP; γ-glutamyl trans peptidase; BUN, blood urea nitrogen; CRP, C-reactive protein; ANA, anti-nuclear antibody; ANCA, anti-neutrophil cytoplasmic antibody; APA, antiphospholipid antibody.

Test	Value	Normal range	Test	Value	Normal range
WBC	8400 /μL	3100-8400	BUN	18 mg/dL	8-20
Hb	12.0 g/dL	11.4-14.6	Creatinine	0.61 mg/dL	0.46-0.79
Plt	10.3×10^3^ /μL	15-40	Na	135 mmol/L	135-145
PT-INR	1.23	0.85-1.15	K	2.6 mmol/L	3.5-4.5
APTT	39.4 sec	25-40	Cl	98 mmol/L	97-106
D-dimer	56.3 μg/mL	<1.0	CRP	34.3 mg/dL	<0.3
AST	83 U/L	13-33	Glucose	129 mg/dL	70-140
ALT	27 U/L	8-42	ANA	(-)	
γ-GTP	30 U/L	<65	P/C-ANCA	(-)	
Total protein	6.5 g/dL	6.5-8.0	SS-A/B antibody	(-)	
Albumin	3.1 g/dL	3.5-5.0	APA	(-)	

**Figure 1:**
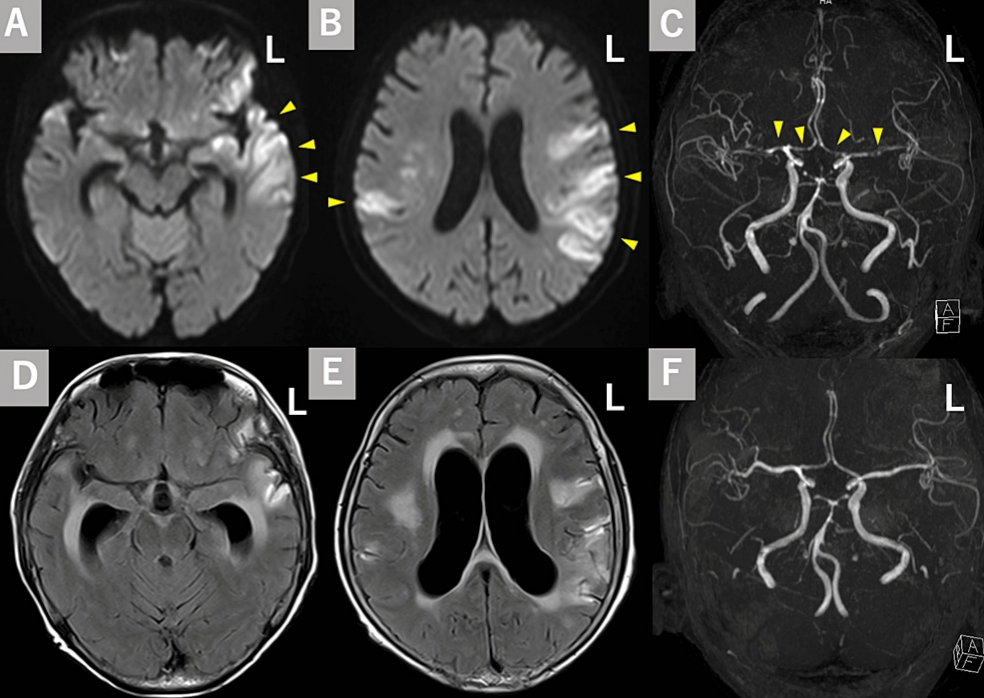
Imaging findings of the present case A, B: Axial diffusion-weighted imaging (DWI) showed hyperintense lesions in the bilateral middle cerebral artery territory (arrowheads). C: Magnetic resonance angiography (MRA) showed multiple stenoses in the bilateral middle cerebral artery, anterior cerebral artery, and basilar artery (arrowheads). D, E: Fluid attenuated inversion recovery (FLAIR) in follow-up showed hydrocephalus. F: Follow-up MRA showed an improvement in arterial stenosis.

Her pneumococcal urinary antigen test was positive. The cerebrospinal fluid (CSF) examination showed marked pleocytosis (57 cells/µL, with polymorphonuclear leukocyte 70%, normal value of cell count ≤5/µL), and elevated protein levels (994 mg/dL, normal value of protein <45-60mg/dL). Blood and CSF cultures revealed penicillin-sensitive *S. pneumoniae* (PSSP). Whole-body computed tomography (CT) showed asplenia and postoperative changes of gastric cancer. Whole-body CT did not show any sinusitis or pneumonia. We diagnosed her with pneumococcal meningitis as OPSI. We started intravenous ceftriaxone and vancomycin. We also started dexamethasone 6.6 mg four times a day for four days. After starting initial therapy, her consciousness disturbance gradually improved (Glasgow coma scale of 13; E4V4M5). Her fever and inflammatory response also improved. Therefore, we discontinued dexamethasone on day 5. Since CSF culture revealed PSSP on day 5, vancomycin was also discontinued on day 6 and we continued only ceftriaxone. However, on day 10, consciousness disturbance (Glasgow coma scale of 7; E1V2M4), and right hemiparesis appeared. Brain magnetic resonance imaging (MRI) revealed acute cerebral infarctions in the bilateral middle cerebral artery territory (Figures 1A, 1B). Magnetic resonance angiography (MRA) showed multiple intracranial artery stenosis in the bilateral middle cerebral artery, anterior cerebral artery, and basilar artery (Figure 1C). Although repeated CSF on day 14 examination showed pleocytosis and elevated protein, CSF culture did not detect any bacteria including *S. pneumoniae*. Follow-up MRI on day 27 showed diffuse hydrocephalus (Figures 1D, 1E). Follow-up MRA showed multiple intracranial artery stenoses improvement, which could suggest vasospasm (Figure 1F). Contrast-enhanced MRI showed no perivascular enhanced lesion. Since cerebral vasospasm occurs after five days from steroid withdrawal, and MRI findings and CSF examination did not show recurrence or deterioration of meningitis, or cerebral vasculitis, we hypothesized that remained excessive immune response after steroid withdrawal was associated with delayed cerebral vasospasm and hydrocephalus. After restarting an intravenous corticosteroid and tapering gradually, her consciousness disturbance slightly improved and meningitis remission was archived. Because of severe aphasia and bulbar palsy due to bilateral cerebral infarctions, she was unable to oral intake. She was transferred to the nursing hospital.

## Discussion

This is a case of pneumococcal meningitis as OPSI which showed delayed cerebral vasospasm as a steroid rebound phenomenon. This case would alert the clinician to the dexamethasone tapering methods in pneumococcal meningitis as OPSI. 

Pneumococcal meningitis sometimes causes neurological complications such as cerebral infarction, vasospasm, and hydrocephalus [3]. The causes of these neurological complications are complicated and reportedly caused by the excessive immune response against bacterial microstructures, direct pneumococcus invasion, or cerebral vasculitis [5,6]. Dexamethasone can suppress excessive immune responses to bacterial microstructures by suppressing inflammatory cytokines such as IL-1 and TNF-α in spinal fluid [8]. For the prevention of neurological complications, dexamethasone (0.15 mg/kg/day) for four days has been recommended [4]. The steroid rebound phenomenon which occurs as dexamethasone withdrawal has been reported in pneumococcal meningitis [5,6,7]. Hydrocephalus could also occur accompanied by the rebound effect [7]. Its mechanism is characterized by vascular complications caused by the reactivation of the primary inflammatory response by bacterial fragments [5,6]. The inflammatory infiltrates surrounding the cerebral vasculature in the inflated subarachnoid space lead to vasospasms and thromboses of arteries and veins, and subsequent focal cerebral ischemia [9]. To avoid the rebound effect, a gradual dexamethasone withdrawal rather than the abruptly terminated four-day course, or longer treatment with progressive tapering is advocated [5,6]. 

In the present case, the initial treatment including dexamethasone appeared to be successful and was used for four days. However, the patient developed delayed cerebral vasospasm after dexamethasone withdrawal. Since the follow-up CSF examination did not detect *S. pneumoniae*, infection recurrence would not be considered. Cerebral vasculitis was another differential diagnosis. However, there were no findings suggestive of vasculitis in the additional blood test and contrast-enhanced MRI. We considered the lower possibility of cerebral vasculitis. 

In the present case, the background of asplenia would be especially important. The spleen plays an important role in the removal of capsular-bearing bacteria such as *S. pneumoniae* [2] and asplenia often leads to inadequate bacterial removal. Therefore, it would be speculated that the immune response to the bacterial microstructure would be more long-lasting in asplenia patients. Considering the present case was asplenia and the usual dexamethasone use could not adequately suppress the immune response to bacterial microstructures, it was thought that the cerebral vasospasm due to steroid rebound phenomenon by residual inflammation was more likely to occur rather than cerebral vasculitis. Since the pathological findings could not be obtained in the present case, complete differentiation from vasculitis was difficult. Further case accumulation would be essential for the precise pathophysiology of steroid rebound phenomenon.

There have been no reports regarding the delayed cerebral vasospasm as steroid rebound phenomenon in pneumococcal meningitis as OPSI. Since pneumococcal meningitis as OPSI has a higher risk of neurological complications, clinicians should consider longer and more cautious steroid tapering.

## Conclusions

The present case was delayed cerebral vasospasm after dexamethasone withdrawal in a patient with pneumococcal meningitis as OPSI. Pneumococcal meningitis as OPSI has a higher risk of neurological complications and is sometimes fatal. Especially, asplenia often leads to inadequate bacterial removal. Therefore, it would be speculated that the immune response to the bacterial microstructure would be more long-lasting in OPSI. Since the steroid rebound phenomenon could occur after dexamethasone withdrawal, clinicians should consider longer and more cautious dexamethasone tapering, especially in pneumococcal meningitis as OPSI.
